# Macrophages immunomodulation induced by *Porphyromonas gingivalis* and oral antimicrobial peptides

**DOI:** 10.1007/s10266-023-00798-w

**Published:** 2023-03-10

**Authors:** Blanca Esther Blancas-Luciano, Jaime Zamora-Chimal, Pablo Gomes da Silva-de Rosenzweig, Mariana Ramos-Mares, Ana María Fernández-Presas

**Affiliations:** 1 Departamento de Microbiología y Parasitología, Facultad de Medicina, Col. Universidad Nacional Autónoma de México, Av. Universidad 3000, CP 04510 Mexico City, Mexico; 2 Posgrado en Ciencias Biológicas, Unidad de Posgrado, Ciudad Universitaria, Edificio D, 1° Piso, Mexico City, Mexico; 3 Unidad de Investigación en Medicina Experimental, Universidad Nacional Autónoma de México, Hospital General de México, Dr. Balmis, 148 Col. Doctores, Del. Cuauhtémoc, C.P. 06726 Mexico City, Mexico; 4 Centro de Investigación en Ciencias de la Salud (CICSA), FCS, Universidad Anáhuac México Campus Norte, Huixquilucan, State of Mexico Mexico

**Keywords:** Periodontitis, Macrophages, Oral antimicrobial peptides, Immune response

## Abstract

*Porphyromonas gingivalis* is a keystone pathogen associated with periodontitis development, a chronic inflammatory pathology characterized by the destruction of the supporting teeth structure. Macrophages are recruited cells in the inflammatory infiltrate from patients with periodontitis. They are activated by the *P. gingivalis* virulence factors arsenal, promoting an inflammatory microenvironment characterized by cytokine production (TNF-α, IL-1β, IL-6), prostaglandins, and metalloproteinases (MMPs) that foster the tissular destruction characteristic of periodontitis. Furthermore, *P. gingivalis* suppresses the generation of nitric oxide, a potent antimicrobial molecule, through its degradation, and incorporating its byproducts as a source of energy. Oral antimicrobial peptides can contribute to controlling the disease due to their antimicrobial and immunoregulatory activity, which allows them to maintain homeostasis in the oral cavity. This study aimed to analyze the immunopathological role of macrophages activated by *P. gingivalis* in periodontitis and suggested using antimicrobial peptides as therapeutic agents to treat the disease.

## Introduction

Oral health depends on the homeostatic balance between the host immune response and the microbiota of the oral cavity, constituted by approximately 700 microbial species [1, 2]. Biological and non-biological surfaces of the oral cavity are covered by antimicrobial film—for instance, the periodontal biofilm could be a source for dissemination and development systemic infections, and for this reason, the regulation of this balance, between the host and oral microorganisms, is essential for the maintenance of this homeostasis in the oral cavity [3]. The disequilibrium of this balance induces dysbiosis and inflammation that can lead to periodontal diseases [4]. Periodontal diseases are a group of inflammatory pathologies with a high worldwide incidence and prevalence [5]. Periodontitis is a prevalent disease, with an inflammatory infectious etiology, of tooth supporting tissue, whose etiopathogenesis is linked to an imbalance between oral microbiota and host’s response [6]. Periodontal disease is classified + by The Global Burden of Disease as the 11th chronic disease with the highest prevalence worldwide [7]. The World Health Organization (WHO) has reported that it affects 10–15% of the world's population [8, 9]. However, the American Academy of Periodontology (AAP) reported that 70.1% of the American population over 65 years courses with this disease and a prevalence of 67.2% indicated has been in the Mexican population [10, 11].

Periodontitis leads to tooth loss due to a chronic inflammatory process that destroys the support tissues (gingiva, periodontal ligament, radicular cement, and alveolar bone) [12, 13]. In the inflammatory infiltrate of these lesions, *Porphyromonas gingivalis* is the etiological agent of the most severe forms of the disease [4]. This bacterium expresses diverse virulence factors that interact with cellular populations, including epithelial and endothelial cells, neutrophils, fibroblasts, and macrophages [14, 15]. Macrophages participate in the tissue repair process and the defense against microorganisms [16]. However, *P. gingivalis* can persist intracellularly in macrophages and induce effector mechanisms that contribute to the inflammatory response in periodontal disease [17, 18]. Additionally, macrophage depletion in the periodontitis murine model induced by *P. gingivalis* showed a low-grade chronic inflammation and periodontal tissue destruction compared with wild-type animals [18], demonstrating the central role of macrophages in the immunopathology induced by the bacterium.

Saliva is a natural defense mechanism that produces antimicrobial molecules that protect oral tissues from the proteolytic and inflammatory activity caused by the virulence factors of *P. gingivalis* [19, 20]. These molecules are named oral peptides because they are produced by the salivary glands and oral epithelium [21]. The presence of 45 peptides has been reported in human saliva [22], some of them can interact with the macrophages present on the surface of oral lesions, inducing their stimulation, chemotaxis, phagocytosis, and regulation of the inflammatory process [23, 24]. In this review, we analyze the role of macrophages during *P. gingivalis* infection and the possible immunomodulatory role of oral antimicrobial peptides.

## *P. gingivalis* in periodontitis

Two main groups of periodontal disease have been described: gingivitis and periodontitis. Gingivitis is considered the initial inflammatory stage of gums, limited to the soft adjacent tooth tissues [25]. On the other hand, periodontitis is considered a chronic inflammatory stage of the periodontal support tissues [26]. The development of both pathologies is associated with the presence of a dysbiotic subgingival microbial biofilm [27], composed of the red complex, which groups periodontopathogenic microorganisms like *Treponema denticola*,* Tannerella forsythia*, and *P. gingivalis* [28]. Recent studies have reported that *P. gingivalis* has been isolated in 75.8% of the periodontal pockets of patients with periodontitis [29]. In murine models, this bacterium can induce experimental periodontitis [30]. *P. gingivalis* is a Gram-negative rod-shaped, anaerobic, strictly facultative, and asaccharolytic bacterium. In blood agar plates, it forms black pigment colonies that contain a heme-group on the cellular surface, which comes from hemoproteins, gingival crevicular fluid, and erythrocytes. For these factors, the growth of *P. gingivalis* is conditioned in iron and vitamin K-rich nutritional complexes [8, 31, 32].

Inside the oral environment, the bacterium finds its ideal niche in the subgingival sulcus within three microenvironments: the radicular surface of the tooth, gingival crevicular fluid, and the gingival epithelium [14]. Survival in these microenvironments depends on the expression of its virulence factors: cysteine proteases (gingipains), hemagglutinins, lipopolysaccharide (LPS), nucleoside diphosphate kinase (NDK), and fimbriae [33, 34, 35]. Recent studies have shown that LPS, gingipains, and fimbriae are the most important pathogenic molecules that contribute to the establishment of *P. gingivalis* [34].

During the early stages of *P. gingivalis* infection, the gingival epithelial cells recognize these virulence factors of the bacterium through Toll-like receptors (TLRs), NOD (NLR), and lectin receptors (CLRs). The interaction of these receptors with virulence factors induces inflammatory cytokines, like IL-1β, IL-6, and TNF-α in these cells [34, 36]. The presence of inflammatory cytokines and the persistence of *P. gingivalis* in the oral cavity induce recruitment of monocytes and infiltration of macrophages toward the injury site [37, 38, 39]. This inflammatory environment generates subsequent pathological conditions, including metalloproteinases production, responsible for the degradation of the connective tissue of the gum and the periodontal ligament [40, 41], and an unbalanced production of osteoblasts and osteoclasts, which leads to the reabsorption of the alveolar bone, eventually bringing about tooth loss [42].

## Importance of macrophages in periodontitis

The macrophages population comprises 5–30% of the cells recruited in the inflammatory infiltrate of patients with periodontitis [43]. These cells are the main source of IL-1β, TNF-α, IL-6, and IL-8, which are the cytokines responsible for the migration and activation of cells like neutrophils and lymphocytes to the inflammatory infiltrate [41, 44, 45]. The relevance of macrophages in the periodontal disease has been demonstrated in a murine model of periodontitis using mice infected with *P. gingivalis* for 58 days. In this work, F4/80 + macrophages were increased four-times in the gum. Depletion of these cells with clodronate liposomes reduced the levels of bone resorption compared to the controls without treatment [37]. These data suggest that macrophages are the main responsible for the development of bone loss in periodontitis.

Macrophages play distinct roles during periodontitis stages, the chronic phase is associated with the presence of M1 and M2 phenotypes in the inflammatory infiltrate, being the M1 macrophages the most abundant [46, 47]. Macrophages arrive at the inflammatory infiltrate and are differentiated into M1 macrophages by the presence of cytokines like IFN-γ and TNF-α [48], which are secreted by epithelial cells following bacterial challenge [49]. Another differentiation pathway is through the direct stimulation of the TLRs of the macrophage by the pathogen-associated molecular patterns (PAMPs). For example, the LPS of *P. gingivalis* can induce differentiation toward M1 macrophages [50, 51]. M1 macrophages activation induces inflammatory mediators production, such as IL-1β, IL-6, IL-8, and TNF-α, and the inducible nitric oxide synthase enzyme (iNOS) [52], some of these mediators, such as nitric oxide (NO) and IL-8, amplify the inflammatory response by increasing the local blood flow and the recruitment of leukocytes [53, 54]. Furthermore, IL-1β and TNF-α increase the expression of matrix metalloproteinases (MMPs), like MMP-1, MMP-13, MMP-8, and MMP-9 [40, 55], which have as main substrates collagen I, III, and IV proteins, as well as components of the extracellular matrix, such as fibronectin and tenascin [56]. These data suggest that the inflammatory mediators favor degradation of the extracellular matrix by MMP activation [57].

M2 macrophages play a role in relieving inflammation and inducing tissue repair in periodontitis. They produce anti-inflammatory cytokines, such as IL-10, IL-4, and TGF-β [45]. These cytokines downregulate pro-inflammatory cytokines, MMPs, and the stimulation of osteoblasts inhibiting bone resorption [57]. These events promote tissue regeneration, angiogenesis, restore the inflammatory mediators produced by M1 macrophages, and contribute to regulating the osteoclastogenesis process [58, 59]. Osteoclastogenesis is mediated by the ligand of the activator receptor for the nuclear κB (RANKL) factor, its RANK receptor, and osteoprotegerin (OPG). RANK and RANKL are expressed in the dental follicles during tooth eruption and in the periodontal tissue in the adult age [60]. The activation of RANK-RANKL in osteoclasts activates the transcription of genes related to NFATc1, c-Fos, and NF-κB that modulate differentiation and activation of osteoclasts, inducing bone loss and the subsequent loss of the tooth [61, 62]. OPG is a homeostatic control factor that protects the dental cement and bone against radicular reabsorption [60, 63]. In the periodontal disease, TNF- α and IL-1β produced by M1 macrophages increase RANKL and diminish OPG [52, 64], inducing bone loss. These data add evidence to the notion that the M1 macrophage is highly involved in the loss of bone tissue due to its inflammatory role in periodontal disease.

M2 macrophages play a role in relieving inflammation and inducing tissue repair in periodontitis. They produce anti-inflammatory cytokines, such as IL-10, IL-4, and TGF-β. These cytokines downregulate pro-inflammatory cytokines, MMPs, and stimulate osteoblasts, inhibiting bone resorption. These events promote tissue regeneration, angiogenesis, and restore homeostasis [46]. The injection of M2 macrophages decreases the inflammatory response and osteoclast differentiation in mice infected with *P. gingivalis* compared to the unstimulated control group [65] indicating that the polarization toward the M2 phenotype could favor the regulation of the inflammatory response and protect against bone resorption.

Another role of macrophages in *P. gingivalis* infection is their capacity as host cells. This phenomenon is helped by the capsule from *P. gingivalis*, in which macrophages can phagocyte non-encapsulated ATCC 33277 and encapsulated W83 strains of *P. gingivalis*. However, W83 invaded these immune cells better than all other strains tested, demonstrating that encapsulated strain has a greater invasiveness capacity than the no-encapsulated [17]. It also demonstrated that bacteria survive inside macrophages [8].

These data suggest that the balance of M1 and M2 macrophages could mediate bone resorption due to regulating the inflammatory conditions in periodontitis. Additionally, macrophages can internalize *P. gingivalis*, which can survive inside of these cells.

## Modulation of macrophages by *P. gingivalis* virulence factors

**Fig. 1:**
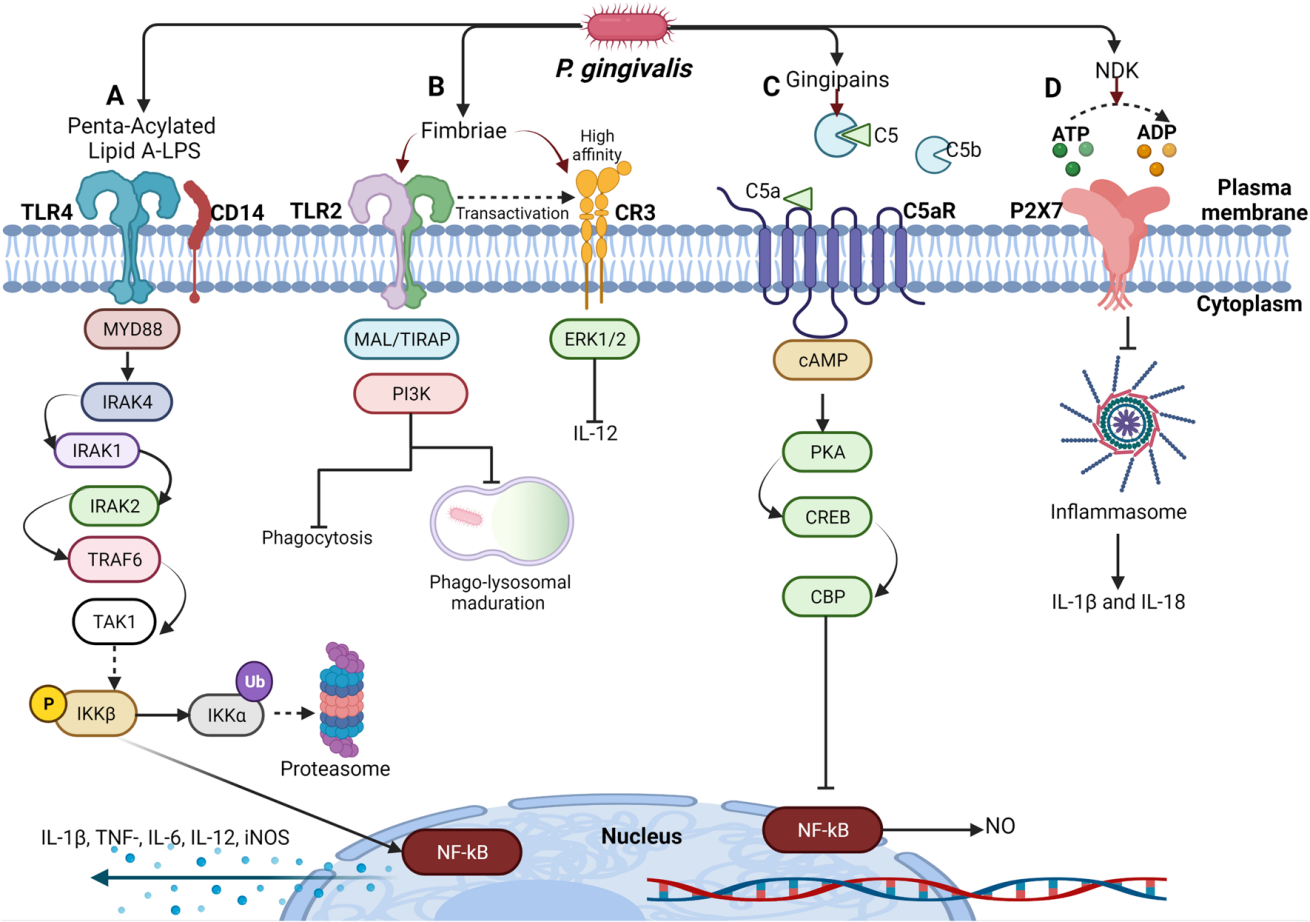
Immunomodulation induced by *Porphyromonas gingivalis* virulence factors on macrophages. **A** Recognition of LPS of *P. gingivalis* by TLR4. The canonical activation of this receptor favors the translocation of NF-κB to the nucleus, inducing the production of inflammatory cytokines. **B** TLR2 recognizes bacterial fimbriae, which induces the production of inflammatory mediators, via MyD88. TLR2 also activates a Mal/TIRAP-dependent pathway with PI3K activation, reducing phagocytosis and phagolysosome maturation. TLR2 activation transactivates CR3, increasing its affinity toward fimbriae. Activation of CR3 induces the ERK 1/2 signaling pathway that reduces IL-12 production. **C** The gingipains of *P. gingivalis* degrade C5 to C5a, increasing cAMP production, which activates PKA, allowing the binding of CREB to CBP, inhibiting the NF-κB. **D** The P2X7 receptor is activated by binding to eATP. NDK hydrolyzes ATPe to ADP, which inhibits P2X7 activation; preventing inflammasome activation and cell death by determining its intracellular survival in macrophages

Virulence factors from *P. gingivalis* are associated with pathogenicity and evasion mechanisms from the host immune response. They favor the persistence of *P. gingivalis* in the oral environment and the subsequent progression to periodontitis [66]. These virulence factors include LPS, gingipains, cysteine proteases, fimbriae, and NKD (Fig. 1), which are the most important virulence factors involved in *P. gingivalis* establishment [34, 67]. These molecules are recognized by PRRs, like TLRs [34]. TLRs induce microbicidal mechanisms for the elimination of pathogens. However, they have been related to the development of pathological conditions caused by the inflammatory process [68]. Case–control studies in adolescent patients with periodontitis revealed the connection between the establishment of the disease and single-nucleotide polymorphism (SNP) in the TLR 1, 4, 7, and 8 genes [69]. This suggests that TLRs and the virulence factors of *P. gingivalis* are responsible for the development of periodontitis. In the following paragraphs, the main *P. gingivalis* virulence factors are described, and their relationship with the immune response mechanisms in macrophages.

### LPS

The main virulence factor of *P. gingivalis* is its LPS, due to its abundance on the bacterial surface and its ability to activate the innate immune system from the host [15]. LPS has heterogeneous and atypical variations that condition its recognition by TLR receptors (Fig. 1). LPS from *P. gingivalis* has two isoforms of lipid A, one tetra-acylated and one penta-acylated, which are expressed depending on the hemin concentration present in the microenvironment inhabited by the bacterium [69, 70]. When there is less amount of hemin, lipid A is penta-acylated and induces activation of the TLR4 receptor. At a higher amount of hemin, lipid A is tetra-acylated and dephosphorylated and acts as an antagonist of the TLR4 receptor [70]. In the blood flow, the LPS of *P. gingivalis* is associated with the lipopolysaccharide-binding protein (LBP). The LBP–LPS complex is subsequently transported to the membrane of macrophages, with CD14 allowing the oligomerization and activation of TLR4 [15].

Recognition of *P. gingivalis* mediated by TLR4 induces the activation of intracellular signaling cascades that activate transcriptional factors like NF-κB [71]. The canonical pathway of this receptor is mediated by the adaptor protein, MyD88, recruited through its TIR domain toward the receptor. Once MyD88 has bound to the TLR4, the cytoplasmic kinase, IRAK4, is recruited and activated, followed by IRAK1 and IRAK2, inducing their interaction with TRAF6, an E3-type ligase that recruits TAK1 complexes [72]. TAK1 phosphorylates Iκκβ, which allows the phosphorylation and degradation of IκBα, promoting the translocation of NF-κB to the nucleus and inducing the production of inflammatory mediators, like IL-1β, IL-6, IL-12, TNF-α, and iNOS [73]. The aforementioned case suggests that LPS is involved in inflammatory cytokines production that contributes to the destruction of the periodontal tissue in *P. gingivalis* infection.

### Fimbriae

The *P. gingivalis* fimbriae are filamentous protein polymers located on the cell surface of the bacterium [74]. This virulence factor is responsible for the colonization and invasion process; besides, it can induce the production of cytokines, such as IL-1β, IL-6, and TNF-α, and metalloproteinases like MMP-9 [71]. TLR2 has been implicated in the regulation of these processes described below.

Macrophages recognize bacterial fimbriae through TLR2, which induces their activation in a MyD88-dependent pathway with subsequent nuclear NF-kB translocation that promotes the production of inflammatory mediators [75]. However, activation of TLR2 by fimbriae also triggers a Myd88-independent pathway, in which TLR2 recruits the Mal/TIRAP adapter protein that interacts with PI3K. It has been shown that the TLR2-PI3K pathway can prevent phagosome–lysosome fusion into macrophages, which reduces the number of intracellular bacteria and promotes the survival of internalized bacteria [76].

In neutrophils, the co-activation of TLR2-C5aR induced by *P. gingivalis* fimbriae has been described. These events decrease phagocytosis through RhoA inhibition, a GTPase that favors actin polymerization [77, 78] (Fig. 1), suggesting that the bacterium can alter phagocytosis, avoiding intracellular microbicidal mechanisms of macrophages. On the other hand, TLR2 activation by *P. gingivalis* fimbriae can induce transactivation of the complement receptor 3 (CR3). This event increases CR3 affinity, allowing *P. gingivalis* fimbriae to interact with this receptor, whose signaling reduces intracellular bacterial death and decreases IL-12 production in macrophages through activation of ERK 1/2 [14, 79].

Fimbriae contribute to the evasion of macrophage’s microbicidal mechanisms due to the TLR2-PI3K activation pathway involved in phagosome–lysosome fusion, which could favor *P. gingivalis* survival.

### Cysteine proteases or gingipains

Cysteine proteases are extracellular structures of *P. gingivalis* responsible for the proteolytic activity of the bacterium [80, 81]. Based on the specific substrate, they are divided into arginine-dependent Rgp cysteine proteases (RgpA, RgpB) and lysine-dependent Kgp cysteine proteases [81]. These proteases contribute to evading complement lysis since they degrade components such as the C3, which prevents the production of the C3b opsonin, the C5 convertase enzymatic complex, and the generation of C5b, which participate in the formation of the membrane attack complex (MAC) on the surface of *P. gingivalis* [82, 83].

Cysteine proteases act analogously to the convertase C5 enzymatic complex of the complement system generating more than 30 nM of C5a in the human serum [34, 84]. In macrophages, C5a binds to the complement receptor, C5aR. The co-incubation of C5a and *P. gingivalis* with macrophages synergizes the production of cyclic adenosine 3′,5′-monophosphate (cAMP) (Fig. 1), whose increment activates the protein kinase A (PKA), which promotes the binding to the cAMP response element-binding protein (CREB) and the nuclear coactivator CREB-binding protein (CBP) [85]. This binding suppresses the NF-κB pathway, reducing NO production, and decreasing the bactericidal mechanism of the macrophage [34]. These findings suggest that the generation of C5a by *P. gingivalis* cysteine proteases prevents the complement-mediated lysis leading to the inhibition of macrophage microbicidal mechanisms, such as NO production.

### NDK

*Porphyromonas gingivalis* can secrete NDK, a virulence factor whose mechanism of action consists of catalyzing the hydrolysis of extracellular ATP (eATP) toward ADP, interfering with the activation of the P2X7 receptor in macrophages [33, 86]. The interaction of ATP with the P2X7 receptor in macrophages induces a great variety of cellular events, including cell death, generation of reactive oxygen species, inflammasome activation, and the release of inflammatory cytokines, such as IL-1β and IL-18 [87] (Fig. 1). On the other hand*,* NDK reduces epithelial cell death by phosphorylating heat-shock-protein-27 (HSP27)-associated human gingival epithelial cells. This mechanism blocks mitochondrial cytochrome c release and reduces the activation of caspase 9. Furthermore, it has been shown that NDK reduces staurosporine-induced apoptosis in epithelial cells [88].

These studies suggest that NDK is a multifunctional molecule that contributes to *P. gingivalis* survival. It decreases microbicidal mechanisms, reduces the activation of the inflammasome, and inhibits cell death by apoptosis.

## NO-induced by *P. gingivalis* in periodontitis

Nitric oxide synthases (NOS) produce NO in different tissues. There are three main NOS isoforms in mammals: endothelial NOS3 (eNOS), neuronal NOS1 (nNOS), and an inducible NOS (iNOS or NOS 2). The first two are constitutively expressed in endothelial and neuronal tissue, respectively. In contrast, NOS2 (iNOS) could be induced by bacterial endotoxins or pro-inflammatory cytokines, and has been identified in activated macrophages. The three isoforms catalyze substrates, such as L-arginine and molecular oxygen, for NO generation and L-citrulline [89].

NO is a free radical with bactericidal action, which interacts with thiol groups and superoxide anion (O2–), which favors reactive oxygen and nitrogen species (RONS) production, such as S-nitrosothiols (RSNO), nitrogen dioxide (NO_2_), peroxynitrite (ONOO–), dinitrogen trioxide, and dinitrosyl–iron complexes. These intermediaries are responsible for microbial death, induced by DNA deamination, rupture of DNA, inhibition of DNA repair, protein modification, and lipid peroxidation in the bacterium [90, 91].

Patients with periodontitis have increased NO concentration in the saliva compared to healthy individuals [92]. Reher et al. reported that NO concentrations range from 7.78 to 15.79 μM in the saliva of patients with periodontitis, whereas the healthy individual’s NO range is 5.86 μM. Furthermore, there is a correlation between NO level and the number of teeth with a probing depth [93]. *P. gingivalis* W83 survives to NO levels ranging between 4.9 and 19.2 μM [92], which could favor its survival in the oral cavity, participating in the progression and severity of the disease. This production might be an early host defense mechanism against bacterial biofilm proliferation. However, it is involved in the immuno-pathogenic process.

The resistance to oxidative stress generated by NO is due to several mechanisms. The first mechanism is the PG0893 gene expression, which encodes a hybrid cluster protein (HCP) with oxidoreductase activity. This enzyme catalyzes the reduction of hydroxylamine to form ammonia (NH_3_) and water (H_2_O), which decreases nitric oxide concentrations [94]. The second mechanism consists of the rubrerythrin (Rbr) expression by *P. gingivalis*, a protein that acts as a cytoplasmic peroxidase, reducing hydrogen peroxide (H_2_O_2_) into water [95]. In addition, Rbr protects against reactive nitrogen, which allows the survival of *P. gingivalis* in the host [96]. The third mechanism is the hemin binding to the *P. gingivalis* surface. The hemin-containing pigment is constituted of two iron (III) protoporphyrin IX molecules covalently linked via an oxygen atom that, when interacting with dioxygen, acts as a protective barrier against ROS-mediated death. In addition, binding of the μ-oxodimeric and μ-oxobishaem forms of protoporphyrin IX on the bacterial surface allows the inactivation of H_2_O_2_, protecting the bacterium from oxidative stress [95].

Although high NO levels do not contribute to *P. gingivalis* elimination, they induce a side effect on the periodontal tissue, increasing vasodilation and diminishing platelet aggregation, which can contribute to gingival bleeding, aside from having cytotoxic effects on the surrounding tissue, increasing the severity of the disease [94]. In rats with experimental periodontitis, increases in the expression of iNOS and NO are associated with bone resorption [97]. These data suggest that NO participates in the pathogenesis of periodontitis because it induces cytotoxic effects on the gingival and bone tissue that favor the progression of the disease.

## Conventional treatment and oral antimicrobial peptides

The periodontal disease therapeutics is based on controlling infection by mechanical elimination, affected tissue scaling and root planing [98], and the use of bactericides, like chlorhexidine [94], and antimicrobials, such as metronidazole, which can inhibit *P. gingivalis* growth at a minimal inhibitory concentration (CMI) of 0.5–8 µg/mL [99]. Although metronidazole is effective against extracellular *P. gingivalis*, it cannot penetrate infected cells [100]. Furthermore, the use of metronidazole has the disadvantage of side effects for the host, such as diarrhea, vomiting, metallic taste, headache, and dizziness [101, 102]. The clinical application of chlorhexidine is limited by its bitter taste and the generation of extrinsic stains in the teeth and tongue [103]. It should be noted that both therapeutic approaches are limited only to eliminate the bacterium, leaving aside the resolution of the secondary inflammatory process generated by the infection. It has been reported that the mechanical removal of bacteria and the use of antimicrobials do not significantly reduce the production of IL-1β after treatment [40]. Hence, the development of a treatment that could offer an alternative for the inflammatory process control in the periodontal disease is utterly relevant. The antimicrobial peptides (AMPs) arise as promising molecules due to their microbicidal effect and the immunoregulation exerted by them [104].

AMPs are amphipathic molecules usually short (less than 100 amino acids). They have a cationic and an amphiphilic end, constituted mainly by cationic amino acids, like arginine and lysine, that grant them a net positive charge in the order of + 2 to + 9 [105, 106]. In the oral cavity, AMPs are known as oral antimicrobial peptides and are expressed in the oral epithelium, crevicular fluid, neutrophils, salivary glands, and saliva [107].

Forty-five oral antimicrobial peptides have been described and are grouped into functional families: cationic peptides, bacterial agglutination or adhesion peptides, metallic ion chelating agents, peroxidases, proteases inhibitors, and peptides against cell wall [108]. This diversity is essential to protect the oral cavity from microorganisms, as well as for the regulation of immunomodulatory activities [109]. Therefore, they could be used in therapeutic schemes in periodontal disease.

## Microbiocide and immunomodulatory effect of oral antimicrobial peptides

Oral antimicrobial peptides depict a potent and wide-spectrum antimicrobial activity against bacteria, yeasts, fungi, and viruses [110]. This activity is based on electrostatic interactions with the surfaces of the negatively charged microbial membranes [104]. The AMPs interact with phosphate groups and divalent cations, such as Mg^2+^ and Ca^2+^ of bacterial LPS, leading to alterations in the permeability of the cytoplasmic membrane of the bacterium and eventual lysis of Gram (−) bacteria [106]. Furthermore, AMPs can also insert into the membrane forming pores that alter the integrity of the microbial membrane, fostering peptide interaction with specific intracellular targets and causing DNA fragmentation and inhibition of protein synthesis [111].

The AMPs immunomodulatory capacity has been related to different factors, environmental stimuli, the type of cells, and tissues on which they act. Their function depends on interaction mechanisms with cellular receptors and the concentration of the peptides [104, 112]. This immunomodulation could generate pro-inflammatory responses that contribute to eliminating pathogens. On the other side, antimicrobial peptides induce anti-inflammatory effects limiting the inflammation severity [113].

The immunoregulatory role of AMPs is shown in studies about deficiency or reduction of these molecules, which demonstrate the increase of inflammatory responses. For example, Crohn's disease is associated with β-defensin 2 (hBD2) reduced expression in human enterocytes [114]. LL-37 cathelicidin absence is related to the development of periodontal disease in patients with Kostmann’s syndrome [115]. Additionally, the presence of LL-37 in macrophages infected with *Mycobacterium tuberculosis* decreases the production of TNF-α and IL-17 and reduces IL-10 and TGF-β production [116].

The immunomodulatory capacity of AMPs is mediated by their intracellular uptake through endocytosis processes, direct penetration, or the interaction with cellular receptors that induce the phosphorylation adaptor proteins in intracellular signal transduction [106, 117]. They can bind to chemokine receptor type 6 (CCR6), formyl peptide receptor coupled to protein type 1 (FPRL-1), or amplify the inflammatory response mediated by TLRs [117, 118]. Hemshekhar et al. demonstrated that the activity of LL-37 is due to G-protein-coupled receptors (GPCR) and JNK mitogen-activated protein kinase (MAPK) signaling in human monocytic THP-1 cells. Furthermore, the receptor activation facilitates chemokine and anti-inflammatory cytokine-induced interleukin-1 receptor antagonist (IL-1RA) production [119].

These findings demonstrate that these peptides exhibit antimicrobial and immunomodulatory properties. Both mechanisms could contribute to *P. gingivalis* elimination in the macrophage and decrease the inflammatory response associated with the severity of periodontitis. Therefore, we describe some oral AMPs that could affect the macrophage and participate in the therapy against *P. gingivalis* (Table 1).

**Table 1:** Antimicrobial and immuno-regulatory activity of oral antimicrobial peptides against *P. gingivalis*

Antimicrobial peptide	Total concentration in saliva	Antimicrobial activity	Immuno-regulatory activity	Concentration versus *P. gingivalis*	References
Histatin-5	53 µg/mL	Binding to hemagglutinin (HagB) of *P. gingivalis*	Decrease in NO, CCL3/MIP, CCL4/MIP-1β, TNF-α, IL-6 and IL-1β production	Unknown	[123, 124]
Cystatin C	0.9 µg/mL	Inhibition of proteolytic activity Induction of structural damage in cell wall and peptidoglycan	Inhibitions of TNF-α, IL-1β and IL-6 production due to blocking NF-κB activation	Unknown	[129, 130, 131, 132, 136]
LL-37	4–6 µM	Neutralization of LPS—*P. gingivalis*	Osteoclastogenesis reduction Decrease in TNF-α production	MIC: > 125 µg/mL^a^	[141, 143, 144, 145]
β-Defensin saliva	0.15 µg/mK–0.31 µg/mL	Membrane pores formation	Inhibits TLR4 activation; reduces cell death	MIC: 42.1 µg/mL	[129, 136, 139]
Lactoferrin	20 µg/mL	Binding to LPS-*P. gingivalis*	Decrease TNF-α, and IL-6 production	MIC:2 mg/mL	[127, 149]
Nal-P 113	Unknown	Membrane pores formation	Inhibits IL-1β and TNF-α production	MIC:320 µg/mL	[152]
Pep-7	Unknown	Membrane pores formation	Decrease in IL-1β y TNF-a production	MBC:1.7 µM/mL^b^	[153]

^a^*MIC* minimal inhibitory concentration

^b^*MBC* minimal bactericide concentration

### Histatins

Histatins are an antimicrobial peptides family produced only in humans and higher primates [118]. They are polypeptides rich in histidine that are secreted by parotid and submandibular glands and released into the saliva where the mean concentration is 53 µg/mL [121]. In this fluid, three types of histatins have been reported; histatin-1, histatin-3, and histatin-5, of which histatin-5 has been shown to have better antimicrobial activity [122].

Histatin-5 exerts antifungal activity against *Candida albicans* by binding to this microorganism and inducing the release of cellular ATP, inhibiting fungus growth in 23–46% [121, 123]. It has been demonstrated that histatin-5 exerts antibacterial activity against *Streptococcus mutans*, with interruptions in the peptidoglycan and the cytoplasmatic membrane, and induces DNA alterations [124]. Additionally, histatin-5 inhibits *P. gingivalis* gingipains and the activity of MMP-2 and MMP-9 [125].

**Fig. 2:**
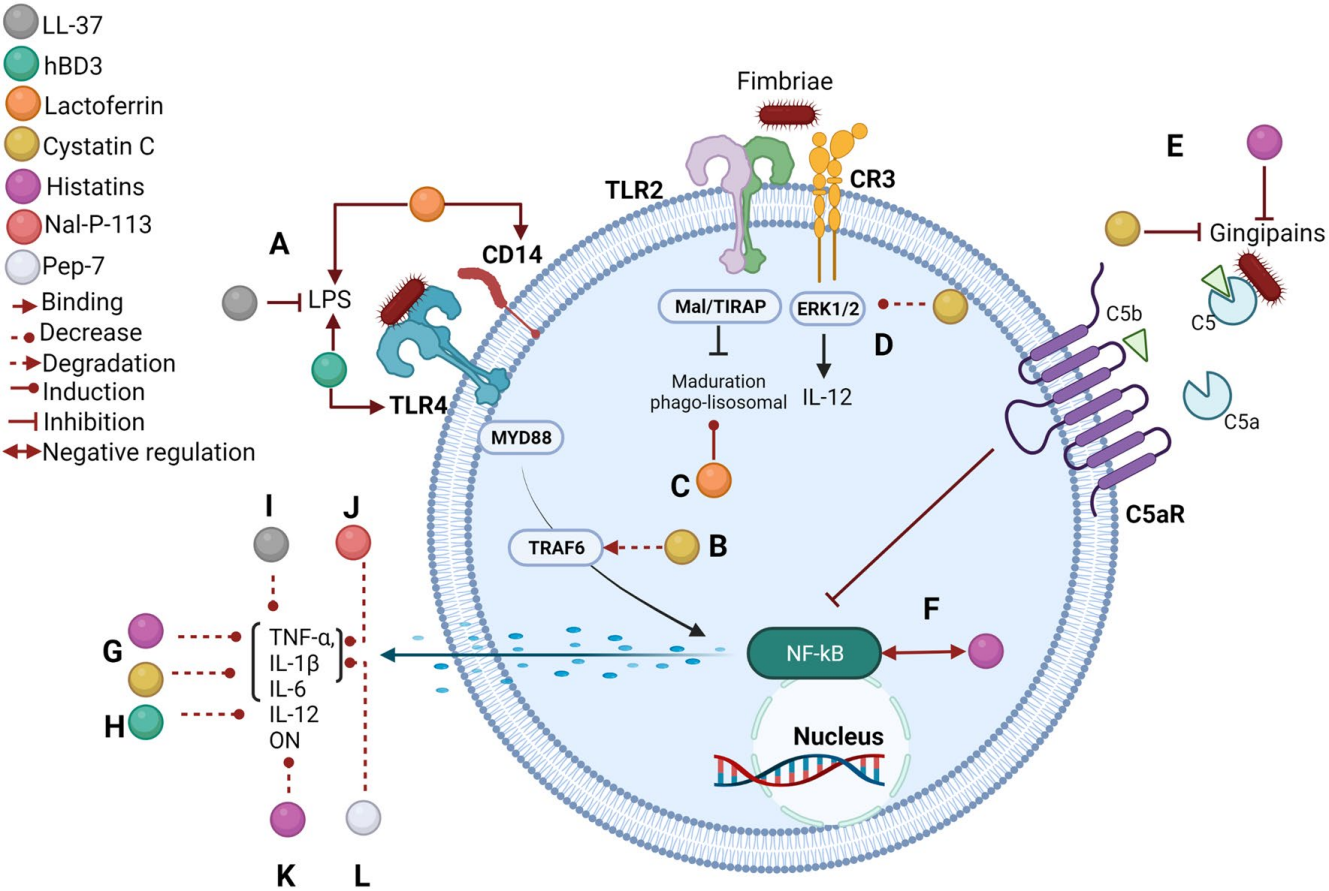
Antimicrobial effect of oral peptides and their possible target in macrophage infected with *Porphyromonas gingivalis.* **A** LL-37 can neutralize at LPS, altering the cell wall of the bacterium. hBD3 and lactoferrin can also bind at this virulence factor. hBD3 also binds to TLR4, blocking its activation, whereas lactoferrin can also bind to CD14, blocking its interaction with this receptor. **B** Cystatin C induces TRAF6 degradation, which decreases inflammatory cytokines production, such as TNF-α, IL-1β e IL-6 **(G)**. **C** Lactoferrin can stimulate phagolysosomal maturation on the macrophage infected with *P. gingivalis*, allowing for bacterial elimination. **D** Cystatin C decreases ERK 1/2 phosphorylation, which could favor IL-12 production in the infected macrophage. **E** Histatin-5 and cystatin C inhibit *P. gingivalis* gingipains. **F** Histatin 1 negatively regulates the NF-κB signaling pathway in infected macrophages, which could decrease the production of NO **(K)** and cytokines, such as TNF-α, IL-1β e IL-6 **(G)**. **H** hBD3, LL-37 **(I)**, Nal-P-113 **(J),** and Pep-7 **(L)** can also decrease inflammatory cytokines production

Histatin-5 has immuno-regulatory activity and inhibits the inflammatory response and tissue damage induced by *P. gingivalis*. It has been demonstrated in silico that binding of the peptide to the hemagglutinin B (HagB) of the bacterium decreases the production of chemokines like CCL3/MIP CCL4/MIP-1β in 49.8% and 39.6%, respectively, as well as TNF-α in 42.4% in dendritic cells [126]. Furthermore, in fibroblasts stimulated with *P. gingivalis* outer membrane proteins, histatin-5 decreased the production of IL-6 and IL-8 by 37% and 47%, respectively, compared to the control without the peptide [127]. Likewise, histatin-1 negatively regulated the JNK and NF-κB signaling pathway in RAW264.7 macrophages, which decreased the production of NO and cytokines, such as IL-6, IL-1β, and TNF-α [128] (Fig. 2).

These results reveal the histatin-5's antimicrobial and immunomodulatory activity that could regulate the inflammatory response in periodontitis, avoiding tissue destruction.

### Cystatin C

This antimicrobial peptide belongs to the type 2 cystatin superfamily, which is ubiquitously distributed in plants, animals, and microorganisms [129]. In humans, it is present in the saliva at 0.9 µg/mL of concentration [130]. One function of cystatin C is cysteine proteases inhibition by binding to their active sites, evading the cleavage of peptide bonds Cystatin C inhibits the proteolytic activity of *P. gingivalis* culture supernatant, decreasing its growth by 50% [131]. Other studies have reported that cystatin C inhibits the growth of *S. mutans* and *Enterococcus faecalis*, exhibiting ultrastructural damage in their cell walls, peptidoglycan breaks, and a decrease in the electron density of the cytoplasm [132].

Some studies have demonstrated that Ds-cystatin, a homologous molecule of cystatin C, isolated from the *Dermacentor silvarum* tick, internalizes into mouse macrophages stimulated with LPS from *Borrelia burgdorferi*, inducing a decrease in inflammatory cytokines, such as IL-1β, IFN-γ, TNF-α, and IL-6. This decrease is mediated by the degradation of TRAF6, which prevented the phosphorylation of IκBα and the subsequent nuclear transport of NF-κB, decreasing the inflammatory response induced by the bacterium [133]. Additionally, it has been reported that human cystatin C internalizes through endocytosis, diminishes phosphorylation of the ERK 1/2 in human monocytes, and decreases IL-1β and TNF-α production in human peripheral blood mononuclear cells stimulated with LPS [134].

The immunoregulatory activity of this peptide has also been tested in other models. *Schistosoma japonicum*-secreted cystatin C induces the polarization of the M2 macrophage, favoring the production of anti-inflammatory cytokines, such as IL-10 and TGF-β. In addition, the adoptive transfer of this cell phenotype favored survival and improved the systemic clinical manifestations of sepsis in a mouse model [135].

These findings show that, in addition to the antimicrobial effect, cystatin C regulates inflammatory mechanisms and polarizes into alternatively activated M2 macrophages, suggesting the therapeutic use of cystatin C against *P. gingivalis* to prevent further damage by periodontitis (Fig. 2).

### Defensins

Defensins are a cationic and amphipathic family of small peptides (2–5 kDa), classified into the α and β subfamilies in humans [136, 137]. Neutrophils and epithelial cells produce salivary α- (HNP1, HNP2, HNP3,) and β-defensins (hBD1, hBD2, hBD3), found at a concentration of 2.7–8.6 µg/mL and 0.15–0.31 µg/mL, respectively [21, 129]. Their antimicrobial effect is based on the peptide integration into the bacterial membranes that results in transmembrane pores formation and the subsequent rupture of the membrane, which leads to the destruction of the bacterium [138].

The hBD3 defensin can bind to LPS and TLR4, in the extracellular space of macrophages incubated with the *Escherichia coli* glycolipid, this blocks TLR4, reducing the activity of MyD88, TRIFF, and NF-κB, and the production of TNF-α, IL-12p40, and IL-6 [139, 140]. Furthermore, hBD3 also suppresses neutrophils apoptosis, acting on the CCR6 chemokine receptor that induces negative regulation in the Bid pro-apoptotic protein, as well as alterations in the mitochondrial membrane potential and caspase 3 activity [141].

In addition, hBD3 suppresses the NF-kB signaling pathway induced by *P. gingivalis* LPS on murine macrophages and RAW264.7 cells. This inhibition decreases the expression of iNOS mRNA and the production of cytokines, such as MCP-1, IL-6, and TNF-α. Additionally, hBD3 induces the expression of Arg 1 mRNA [142]. These findings indicate the role of this peptide in macrophage polarization toward the M2 phenotype and its participation in the anti-inflammatory response in *P. gingivalis* infection. These data suggest that hBD3 defensin could kill *P. gingivalis* and block the interaction of its LPS with TLR4, which could reduce inflammation and exert a protective role in periodontitis (Fig. 2).

### LL-37

Cathelicidin LL-37 is a peptide secreted by neutrophils, found in the saliva at a concentration that ranges from 4 to 6 µM [143]. The cationic and amphipathic LL-37 structure neutralizes the LPS anionic glycolipid, altering the cell wall of Gram-negative bacteria, favoring their death [144, 145]. In macrophages stimulated with LPS purified from *Salmonella typhimurium* and *E. coli*, LL-37 inhibits TNF-α production [146]. In addition, it inhibits osteoclastogenesis processes by inhibiting the translocation of NFAT2 which reduces the formation of osteoclast progenitor cells [147].

These data provide evidence that LL-37 could have antimicrobial activity against *P. gingivalis*; furthermore, participating in the reduction of the inflammatory and osteoclastogenesis process characteristic of periodontitis (Fig. 2).

### Lactoferrin

Lactoferrin is an iron-binding glycoprotein present in the saliva. It can bind to LPS from *P. gingivalis* and CD14, interfering with the formation of the CD14-LPS complex and downregulating the TLR4 signaling pathway [147, 148]. Additionally, the gene polymorphisms of this peptide have been associated with the development of periodontitis in a Taiwanese population [149]. Studies in infected macrophages with *Mycobacterium avium* incubated with lactoferrin showed reduced intracellular bacterial growth. Lactoferrin also enhanced the antimicrobial activity of ethambutol in human macrophages, promoting phagosomal maturation and inflammatory cytokine production, such as TNF-α and IL-6, which foster host resistance to infection [150].

These data suggest that lactoferrin is a key mediator for the inflammatory process control in periodontitis, due to its microbicidal mechanisms against *P. gingivalis* (Fig. 2).

### Nal-P-113 and Pep-7

Nal-P-113 is a cationic peptide rich in histidine derived from histatin-5 [151, 152]. This peptide at a concentration of 320 µg/mL induces cell death in *P. gingivalis* Furthermore, in *P. gingivalis*-induced periodontitis in a rat model, a decrease of IL-1β and TNF-α production and inhibition of bone loss was observed after incubating *P. gingivalis* with this peptide [153].

Pep-7 inhibits the growth of two *P. gingivalis* strains, ATCC 33277 and ATCC 53978 (wp50), at a minimal inhibitory concentration (MIC) of 1.7 µM. Besides, at this concentration, the production of inflammatory cytokines, like IL-1β and TNF-α, was not observed in human gingival fibroblasts [154, 155].

Both peptides display the ability to modulate the inflammatory responses and exhibit remarkable antimicrobial activity. However, further studies are required to elucidate the immunoregulatory mechanism that allows these peptides to modulate macrophage function (Fig. 2).

## Conclusion

The macrophages’ interaction with the periodontal pathogen *P. gingivalis* is a determining factor for periodontitis immunopathology. This pathogen has evolved several mechanisms to evade the host immune system, which are determined by its arsenal of virulence factors, disrupting the signaling pathways of inflammatory cytokines, and leading during chronic infection to the destruction of periodontal tissue. However, oral peptides could act as macrophage regulators and control the inflammatory process, contributing to *P. gingivalis* elimination. Therefore, their use in therapeutic regimens could be promising against periodontitis.

## Abbreviations

*P. gingivalis*: *Porphyromonas gingivalis*
MMPs: Metalloproteinases
LPS: Lipopolysaccharide
NDK: Nucleoside diphosphate kinase
TLRs: Toll-like receptors
NO: Nitric Oxide
iNOS: Inducible nitric oxide synthase enzyme
RANKL: Receptor activator of nuclear-κ B ligand
OPG: Osteoprotegerin
AMPs: Antimicrobial peptides
MIC: Minimum inhibitory concentration
